# Antibiotic tigecycline inhibits cell proliferation, migration and invasion via down‐regulating CCNE2 in pancreatic ductal adenocarcinoma

**DOI:** 10.1111/jcmm.15086

**Published:** 2020-03-06

**Authors:** Jie Yang, Zhen Dong, Aishu Ren, Gang Fu, Kui Zhang, Changhong Li, Xiangwei Wang, Hongjuan Cui

**Affiliations:** ^1^ State Key Laboratory of Silkworm Genome Biology Institute of Sericulture and Systems Biology Southwest University Beibei, Chongqing China; ^2^ Cancer Center Medical Research Institute Southwest University Beibei, Chongqing China; ^3^ Engineering Research Center for Cancer Biomedical and Translational Medicine Southwest University Beibei, Chongqing China; ^4^ Chongqing Engineering and Technology Research Center for Silk Biomaterials and Regenerative Medicine Southwest University Beibei, Chongqing China; ^5^ College of Stomatology Chongqing Medical University Chongqing China; ^6^ Department of Urology Carson International Cancer Center Shenzhen University General Hospital & Shenzhen University Clinical Medical Academy Center Shenzhen University Shenzhen China

**Keywords:** CCNE2, cell proliferation, chemosensitivity, pancreatic ductal adenocarcinoma, tigecycline

## Abstract

Recently, many researches have reported that antibiotic tigecycline has significant effect on cancer treatment. However, biomedical functions and molecular mechanisms of tigecycline in human pancreatic ductal adenocarcinoma (PDAC) remain unclear. In the current study, we tried to assess the effect of tigecycline in PDAC cells. AsPC‐1 and HPAC cells were treated with indicated concentrations of tigecycline for indicated time, and then, MTT, BrdU and soft agar assay were used to test cell proliferation. The effect of tigecycline on cell cycle and cellular apoptosis was tested by cytometry. Migration and invasion were detected by wound healing assay and transwell migration/invasion assay. Expressions of cell cycle‐related and migration/invasion‐related protein were determined by using Western blot. The results revealed that tigecycline observably suppressed cell proliferation by inducing cell cycle arrest at G0/G1 phase and blocked cell migration/invasion via holding back the epithelial‐mesenchymal transition (EMT) process in PDAC. In addition, tigecycline also remarkably blocked tumorigenecity in vivo. Furthermore, the effects of tigecycline alone or combined with gemcitabine in vitro or on PDAC xenografts were also performed. The results showed that tigecycline enhanced the chemosensitivity of PDAC cells to gemcitabine. Interestingly, we found CCNE2 expression was declined distinctly after tigecycline treatment. Then, CCNE2 was overexpressed to rescue tigecycline‐induced effect. The results showed that CCNE2 overexpression significantly rescued tigecycline‐inhibited cell proliferation and migration/invasion. Collectively, we showed that tigecycline inhibits cell proliferation, migration and invasion via down‐regulating CCNE2, and tigecycline might be used as a potential drug for PDAC treatment alone or combined with gemcitabine.

## 1 INTRODUCTION

Pancreatic ductal adenocarcinoma (PDAC) is a highly aggressive malignancy in the digestive tract with a poor prognosis. In the past decade, the treatment of PDAC had yet to be improved.^1^ PDAC accounts for only 3% of all cancers, but is responsible for about 7% of cancer deaths.^2^ Meanwhile, PDAC is the sixth leading cause of death from malignant diseases in China and the third leading cause of death induced by cancers in the USA.^3^, ^4^ Because of the silent nature of PDAC, it is usually only diagnosed when it has highly aggressed and reached as a high‐stage cancer.^5^ The overall five‐year survival rate is less than 5%, and most patients present with developed tumours which are inoperable and non‐curable.^6^, ^7^ Although the current treatments such as chemotherapy, radiotherapy, and surgery are used, the prognosis of patients with PDAC has not improved.^8^, ^9^, ^10^ Considering these, it is meaningful to explore the role of some new drugs with high efficiency for the treatment of PDAC and less toxic side effects on normal cells.

Previous studies have shown that antibiotics have potential value in treating tumours.^11^, ^12^ Tigecycline, a new member of antibiotics with broad spectrum, inhibits protein translation by binding to the 30S ribosomal subunit, thereby blocking charged aminoacyl‐tRNAs to enter into the A‐site of the ribosome and preventing peptide elongation during prokaryotic translation in prokaryotes.^13^ In June 2005, it was approved by the USA Food and Drug Administration (FDA) for the treatment of infections caused by multiple bacteria.^14^

Interestingly, recent studies also show that tigecycline is a promising candidate drug for the treatment of cancers.^15^, ^16^ Tigecycline was firstly shown to have an anti‐cancer effect in human acute myeloid leukaemia (AML) via inhibiting mitochondrial translation.^17^ Then, we found that tigecycline also plays important roles in various solid tumours, such as gastric cancer,^18^ oral squamous cell carcinoma (OSCC),^19^ malignant melanoma,^20^ neuroblastoma 21 and glioma.^22^ Meanwhile, other groups also found that tigecycline acts as a promising anti‐cancer drug for many other kinds of cancers, including triple‐negative breast cancer (TNBC), diffuse large B‐cell lymphomas (DLBCLs), cervical squamous cell carcinoma, ovarian cancer, hepatocellular carcinoma, chronic myeloid leukaemia (CML), prostate cancer and lung cancer.^23^, ^24^, ^25^, ^26^, ^27^, ^28^, ^29^, ^30^ Recently, c‐Abl–specific tyrosine kinase inhibitors (TKIs) in combination with tigecycline were shown to be a promising strategy to treat patients with CML.^31^ Although it also has some side effects, such as hypofibrinogenemia,^32^ mitochondrial dysfunction 33 and chronic otitis,^34^ we cannot ignore its important potential promise in anti‐cancer therapy.

Although it seems that tigecycline has significance in inhibiting the cell viability of pancreatic cancer cell line MIA PaCa2 cells,^23^ the exact effect and its model of action remain to be further explored. In this present study, we showed that antibiotic tigecycline blocks cell proliferation, migration and invasion via down‐regulating CCNE2 in PDAC cells. In addition, tigecycline enhances the chemosensitivity of PDAC cells to gemcitabine both in vitro and in vivo. Therefore, tigecycline might be used as a promising new candidate anti‐cancer agent for PDAC treatment alone or in combination.

## 2 MATERIALS AND METHODS

### 2.1 Cell culture

A total of four different human PDAC cell lines (AsPC‐1, Capan‐1, HPAC, BxPC‐3) were purchased from the American Type Culture Collection (ATCC). Human normal pancreatic duct glandular cell line (HPDE) was obtained from the Ontario Cancer Institute.^35^ HPAC and Capan‐1 were cultured in Dulbecco's modified Eagle's medium (DMEM; Gibco, Thermo Fisher Scientific, USA), and other cells were cultured in Roswell Park Memorial Institute‐1640 (RPMI‐1640; Gibco) medium. The medium was supplied with 10% foetal bovine serum (FBS; Gibco) and 1% penicillin‐streptomycin (P/S; Invitrogen, Thermo Fisher Scientific). The cells were cultured in a CO_2_ incubator (SANYO) at 37°C.

### 2.2 Tigecycline treatment

Tigecycline (TIG, molecular formula: C_29_H_39_N_5_O_8_, relative molecular mass: 585.65; Wyeth, Canada) was dissolved in solvent (dimethyl sulphoxide, DMSO). Tigecycline was then used to treat PDAC cell lines and HPDE cell line at indicated concentrations or times. Isomeric DMSO was used as control (less than 0.1%). The cell morphology changes induced by different concentrations of tigecycline were observed under an inverted microscopy (Olympus). Cell numbers were determined by using a haemocytometer.

### 2.3 MTT assay

After tigecycline treatment in cells, MTT assay was used to test the rate of cell proliferation and cellular viability. The PDAC cells and HPDE cells in logarithmic phase were plated in the 96‐well plates at a density of 2 × 10^3^ cells per well, respectively. Then, 200 μL DMEM or RPMI‐1640 that contained tigecycline in different concentration (1, 2, 5, 10, 20 and 40 μmol/L) was added to cells for different time. Isometric DMSO was used as control. Then, 20 μL MTT (5 mg/mL; Sigma‐Aldrich, Merck) per well was added into the plate and incubated at 37°C for 2 hours. Then, the upper medium was removed gently and 150 μL DMSO per well was added to resolve the sediments, and then, the absorbance was detected by a microplate reader (Thermo Fisher Scientific) at 560 nm after shaking for 5 minutes.

### 2.4 BrdU staining

Cell proliferation was detected by BrdU corporation assay. 2 × 10^4^ cells were plated in the 24‐well plates, treated with tigecycline or isometric DMSO for 24 hours and then incubated with 10 μg/mL thymidine analog 5‐bromo‐2‐deoxyuridine (BrdU, Sigma‐Aldrich) for half an hour. Then, cells were washed three times with 1 × phosphate‐buffered saline (PBS) and fixed in 4% paraformaldehyde (PFA) for a quarter of an hour. After that, cells were permeabilized with 0.1% Triton X‐100 for 10 minutes and incubated with 1 mol/L hydrochloric acid for 10 minutes. Then, cells were blocked with 10% goat serum for 1 hour, and anti‐BrdU rat primary antibody (1:200, Abcam) was incubated for 1 hour. Then, cells were incubated with Alexa Fluor^®^ 594 goat anti‐rat IgG secondary antibody (Life technology, Thermo Fisher Scientific) for 2 hours. Before observing by microscopy, cells were stained with nucleolus dye DAPI (4',6‐diamidino‐2‐phenylindole, 300 nmol/L) for 10 minutes. The percentage of BrdU‐positive cells was counted and reckoned by using the GraphPad Prism 7.

### 2.5 Flow cytometry analysis

Cells were cultured into 6‐well plates containing different concentrations of tigecycline and then collected by trypsinization for flow cytometry analysis. Isometric DMSO was used as control. The changes of cell cycle and apoptosis were analysed by flow cytometry as described previously.^36^ Propidium iodide (BD Bioscience), RNase A (Sigma‐Aldrich) and Annexin V‐APC (BD Bioscience) were used for cell staining. BD Accuri C6 flow cytometer (BD) and FlowJo 7 software were used to analyse the cell cycle and apoptosis.

### 2.6 Western blot assay

Cells were harvested and lysed in a strong RIPA Lysis Buffer (Beyotime) with a serine protease inhibitor phenylmethanesulphonyl fluoride (PMSF, Beyotime). Cell lysates were denatured at 100°C for 10 minutes, and protein concentrations were determined by a BCA protein assay kit (Beyotime, Taicang, Jiangsu, China). Then, 10% or 12% sodium dodecyl sulphate polyacrylamide gel electrophoresis (SDS‐PAGE) gel was used to separate proteins with different molecular weights, and the Trans‐Blot Turbo transfer system (Bio‐Rad, USA) was used to transfer proteins onto PVDF membranes (Millipore, USA). After blocking with 5% bovine serum albumin (BSA) at RT for about 2 hours, primary antibodies against Tubulin (1:1000; Proteintech), CDK2 (1:1000, Cell Signaling), CDK4 (1:1000; Cell Signaling), Cyclin E2 (1:1000; Cell Signaling), E‐cadherin (1:1000; Cell Signaling) and β‐catenin (1:1000; Cell Signaling), MMP2 (1:1000; Cell Signaling), Snail (1:1000, Cell Signaling), PARP and cleaved PARP (1:1000; Cell Signaling), caspase‐3 and cleaved caspase‐3 (1:1000; Cell Signaling) were incubated at 4°C overnight, and then HRP‐conjugated secondary antibodies (Life Technology) were used and incubated for 2 hours. Proteins were finally visualized by an ECL Western blot analysis system (Clinx Science) by using the ECL reagent (Beyotime).

### 2.7 Migration, invasion and wound healing assay

The 24‐well transwells (8 μm pore size; Corning, China) were used in migration and invasion assay. For the migration assay, 5 × 10^4^ cells in 200 μL media with 1% serum were added into the transwell, and cells in 500 μL media contained 20% FBS were added in the well of the 24‐well plate. For the invasion assay, the transwell upper membrane was coated by 50 μL Matrigel (BD). All media contained DMSO or 5 and 10 μmol/L tigecycline, respectively. After incubating at 37°C in a CO_2_ incubator for the indicated time, the chambers were taken out and were washed with 1 × PBS for three times, and non‐migrating and invading cells from the upper membrane in the transwell were removed by cotton swabs. The number of cells in more than five randomly chosen microscopic fields per transwell was counted and calculated.

For the wound healing assay, cells were pretreated with medium without serum overnight and then cultured in 6‐well plates with full confluence. Detailed protocol was performed as previously reported.^20^ All media contain DMSO or 5 and 10 μmol/L tigecycline, respectively.

### 2.8 Quantitative real‐time PCR (qRT‐PCR)

Total RNA was extracted by using RNAiso (TaKaRa, Dalian, China) according to the manufacture's instruction. Then, cDNA was obtained from 2 μg RNA by using the M‐MLV reverse transcriptase (Promega) for each sample. The qRT‐PCRs were performed with the LightCycler 96 real‐time PCR system (Roche) in triplicate. Detailed protocol was performed as before.^37^ The expression of GAPDH was used as control. Primers used were listed as follows:

CDK2‐F: CCAGGAGTTACTTCTATGCCTGA;
CDK2‐R: TTCATCCAGGGGAGGTACAAC;
CDK4‐F: ATGGCTACCTCTCGATATGAGC;
CDK4‐F: CATTGGGGACTCTCACACTCT;
CCNE2‐F: TCAAGACGAAGTAGCCGTTTAC;
CCNE2‐R: TGACATCCTGGGTAGTTTTCCTC;
E‐cadherin‐F: CGAGAGCTACACGTTCACGG;
E‐cadherin‐R: GGGTGTCGAGGGAAAAATAGG;
MMP2‐F: TACAGGATCATTGGCTACACACC;
MMP2‐R: GGTCACATCGCTCCAGACT;
β‐catenin‐F: AAAGCGGCTGTTAGTCACTGG;
β‐catenin‐R: CGAGTCATTGCATACTGTCCAT;
Snail‐F: TCGGAAGCCTAACTACAGCGA;
Snail‐R: AGATGAGCATTGGCAGCGAG;
GAPDH‐F: CACGGATTTGGTCGTATTGGGC;
GAPDH‐R: CTGATTTTGGAGGGATCTCGCC.

### 2.9 Soft agar colony formation assay

Self‐renewal ability of AsPC‐1 and HPAC cells after tigecycline treatment was investigated by the soft agar assay. Briefly, 1 mL medium containing different concentrations tigecycline and mixture of 0.6% agarose were added to each well of the 6‐well plates and waited for its solidification (base agar). Then, the top layer was performed to contain the same as above concentrations tigecycline and mixture of 0.3% agar with 1000 PDAC cells. After incubating at an incubator (SANYO) with 37°C and 5% CO_2_ for 3‐4 weeks, colonies were stained with 200 μL MTT (5 mg/mL) per well and photographed by an EPSON scanner.

### 2.10 Drug combination assay

Tigecycline and gemcitabine were used with 1:1 ratio at 10, 1, 0.1, 0.01 and 0.001 μmol/L to treat 2 × 10^4^ cells cultured in the 96‐well plate, respectively and combinedly. Then, MTT was used to determine cell viability. CompuSyn software (http://www.combosyn.com/) was used to calculate the Combination Index.

### 2.11 Tumour xenografts

One‐month‐old female BALB/C‐nu mice (HUAFUKANG Bioscience) were housed in the specific pathogen‐free room. Human PDAC cell line (AsPC‐1) cells (1 × 10^5^ cells) in 100 μL DMEM were subcutaneously injected into the left flank of mice. After 5 days, tumours were pumped, and the mice were randomly divided into four groups. First group was injected intraperitoneally with 100 μL PBS (contained 0.1% DMSO); the second group was injected intraperitoneally with tigecycline in 100 μL PBS (contained 0.1% DMSO) at 100 mg/kg (mice body weight); the third group was injected intraperitoneally with gemcitabine in 100 μL PBS (contained 0.1% DMSO) at 50 mg/kg; and the fourth group was injected intraperitoneally with tigecycline (100 mg/kg) and gemcitabine (50 mg/kg) in 100 μL PBS (contained 0.1% DMSO). Each group was treated every 2 days for five times. Tumour width and length were measured by a calliper every two days, and tumour volume was calculated by using the formula (volume = tumour length × width^2^ × π/6). Mice weight was also calculated. At the end, tumours were removed from the body of mice, and photographed and weighed. The animal experiment was approved by the Experimental Animal Care and Use Committees of the Institute of Sericulture and Systems Biology and Institutional Animal Care and Use Committees of the Southwest University.

### 2.12 Immunohistochemistry staining

The xenograft tumours were obtained from the mice and were fixed in 4% PFA for 24 hours. Then, the samples were dehydrated by using an ASP300S automatic dehydrator (Leica, Germany), embedded in paraffin, and then deparaffinized, rehydrated and sectioned at 5 μm. The antigen retrieval was performed, and the sections were incubated in 0.3% hydrogen peroxide for 15 minutes and then incubated with the anti‐Ki‐67 primary antibody (1:100, BD Biosciences) at 4°C overnight. Afterwards, the sections were incubated with anti‐rabbit IgG (H + L), biotinylated antibody (Cell Signaling) at RT for 2 hours. The signal was observed under the microscope performed with a DAB reagent (Beyotime). The rates of Ki‐67–positive staining were calculated from five randomly selected fields. In addition, tissues were counterstained with haematoxylin (Sangon).

### 2.13 Vector construction and infection

Human full length CCNE2 (NCBI GenBank No.: NM_057749.2) DNA was obtained by using PCR from 293FT (ATCC) cells, and the DNA fragment was then cloned into PCDH‐CMV‐MCS‐EF1‐puro vector. Then, the CCNE2 overexpression vectors were transfected into 293FT cells by the Lipofectamine 2000 reagent (Invitrogen, USA) to generate viruses. Subsequently, the lenti‐viruses were used to infect human PDAC cells. Detailed protocol would be found in previous report.^37^ The transfected cells were selected with 4 μg/mL puromycin for 108 hours.

### 2.14 Data analysis and statistical methodology

TCGA clinical data were obtained from the R2: Genomics Analysis and Visualization Platform (https://hgserver1.amc.nl). Log‐rank (Mantel‐Cox) test was used in survival analysis, and cut‐off value was determined by using a scanning modus. Two‐tailed unpaired Student's *t* test was performed to show the significance in other studies. *P*‐value <.05 was considered as significant. All the tests were biologically repeated at least three times.

## 3 RESULTS

### 3.1 Tigecycline inhibits cell proliferation in PDAC cells

**Figure 1:**
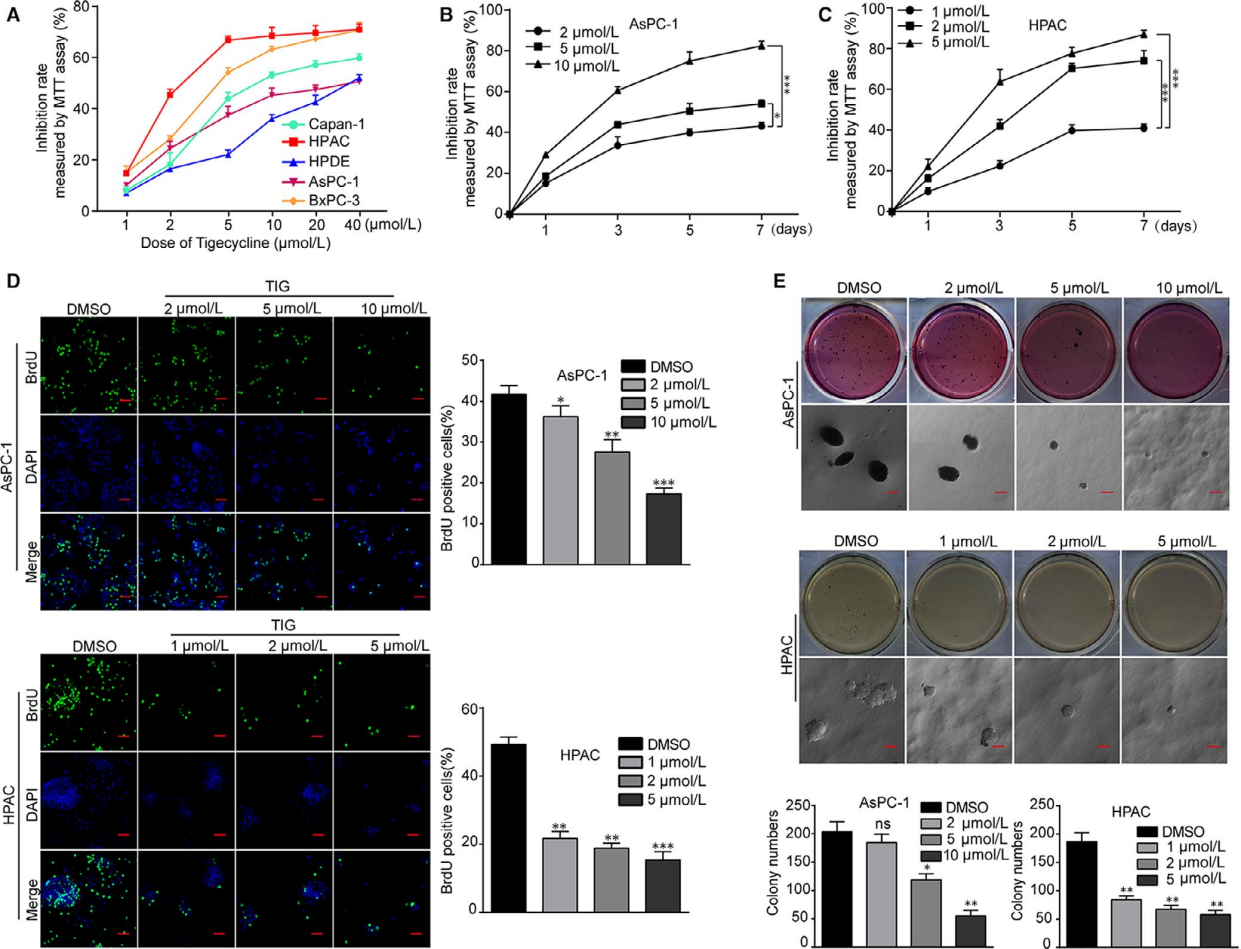
Tigecycline inhibits cell growth and proliferation in human PDAC cell lines. A, Inhibition rates of the four human PDAC cell lines and one human normal pancreatic duct glandular cell line (HPDE) treated with increasing concentrations of tigecycline for 72 h. B, C, Inhibition rates of AsPC‐1 and HPAC cells after treating with different concentrations of tigecycline for the indicated time by using MTT assay. D, Image and quantification of AsPC‐1 and HPAC cells positive for BrdU staining after treating with DMSO or different concentrations of tigecycline for 72 h. Scale bar = 200 μm. E, Colony formation was examined by soft agar assay (1000 cells/well) in AsPC‐1 and HPAC cells after treating with DMSO or different concentrations of tigecycline for 21‐28 d. Colony numbers were counted. DMSO was used as control. Scale bar = 2 mm. All data were shown as the mean ± SD. Student's *t* test was carried out. *P < .05; **P < .01, ***P < .001. ns, not significant. *P*‐value <.05 was considered as statistically significant

The structural formula of tigecycline (TIG) was shown in Figure S1A. In order to investigate the effect of tigecycline on cell proliferation in human PDAC cells, we treated four human PDAC cells (Capan‐1, BxPC‐3, AsPC‐1, HAPC cell lines) and one human normal pancreatic duct glandular cells (HPDE cell line) with different concentrations of tigecycline (1, 2, 5, 10, 20 and 40 μmol/L, isometric DMSO was used as control) for 72 hours in, and then, MTT assay was conducted to detect the inhibition rate. The results revealed that all the four cell lines showed significant inhibition of cell proliferation in a dose‐dependent manner after treatment with tigecycline, while HPDE cell was the most insensitive cell to tigecycline (Figure 1A). Two cell lines including HPAC, which was relatively more sensitive, and AsPC‐1, which was relatively less sensitive, were specially selected for further study. We investigated AsPC‐1 and HPAC cell lines treated with a series of indicated concentrations of tigecycline for 7 days to determine the inhibition rates curve by MTT assay. The results showed a dramatically dose‐dependent decline of cell proliferation in tigecycline‐treated HPAC and AsPC‐1 (Figure 1B, C). Observing by microscopy, AsPC‐1 and HPAC cells showed remarkable morphological changes after treated with applicable concentrations of tigecycline for 72 hours, and cell number was also decreased in a dose‐dependent manner (Figure S1B, C). Furthermore, BrdU assay was implemented. The results showed a notable decrease in the percentage of BrdU‐positive cells in tigecycline‐treated groups, compared with DMSO‐treated groups (Figure 1D). To further confirm the function of tigecycline in self‐renewal capacity, soft agar assay was performed. The results revealed that the colonies were smaller and lesser in cells after treated with tigecycline in a dose‐dependent manner than that of the control groups (Figure 1E). These results showed that tigecycline inhibited cell proliferation of human PDAC cells dose‐dependently.

### 3.2 Tigecycline induces cell cycle arrest at G0/G1 phase in PDAC cells

**Figure 2:**
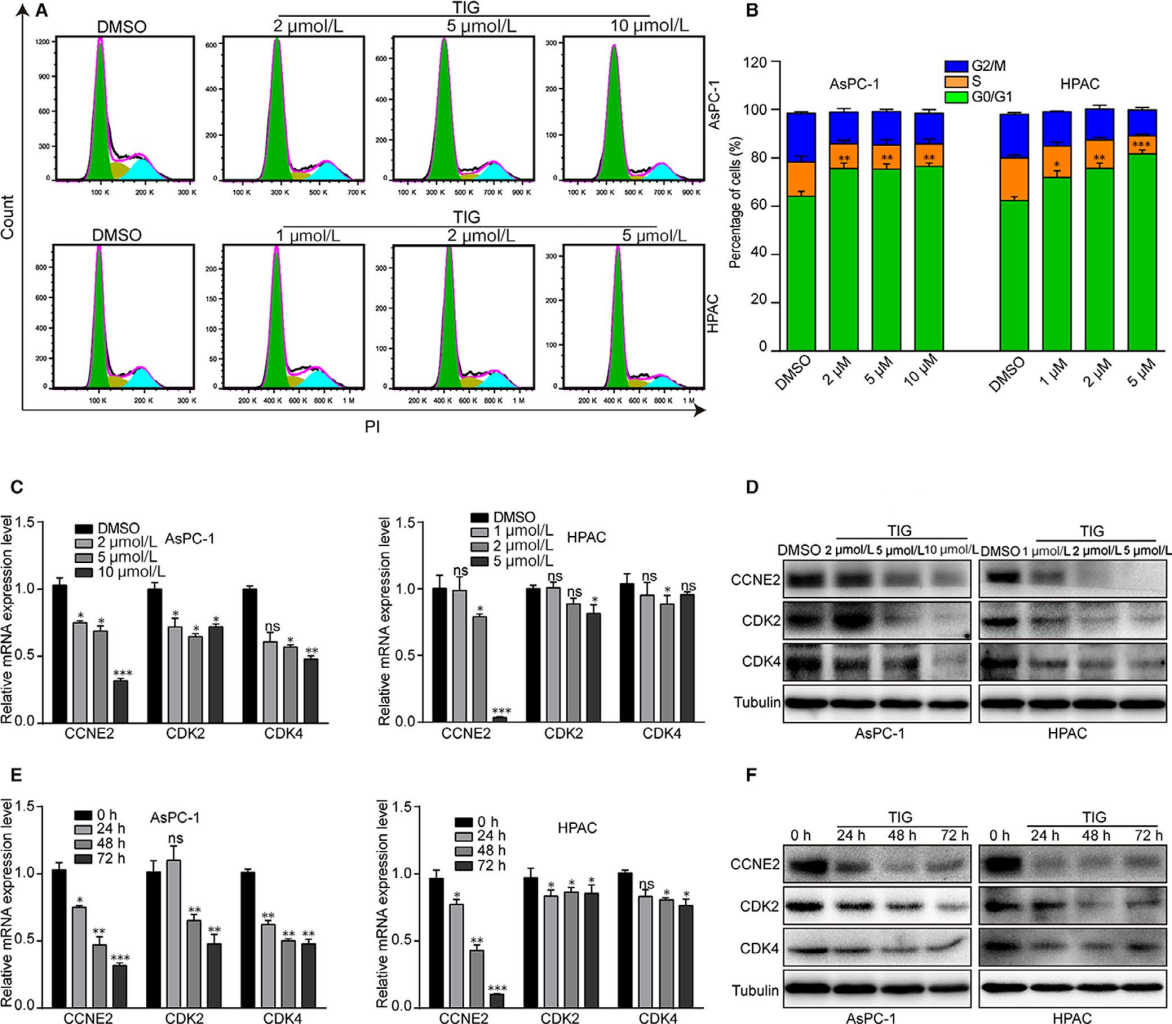
Tigecycline induces human PDAC cell lines cycle arrest in G0/G1 phase. A, B, The cell cycle of AsPC‐1 and HPAC cells treated with different concentrations of tigecycline for 72 h was analysed by flow cytometry. Percentage of indicated AsCP‐1 and HPAC cells in different phases. C, D, The expression of cell cycle‐related mRNA and proteins, CDK2, CDK4 and Cyclin E2 in AsPC‐1 and HPAC cells treated tigecycline in different concentrations for 72 h. Tubulin was used as control. E, F, The expression of cell cycle‐related mRNA and proteins in AsPC‐1 and HPAC cells treated with 5 or 10 μmol/L tigecycline, respectively, in different time for 0, 24, 48 and 72 h. Tubulin was used as control. All experiments were repeated at least three times. All data were used as mean ± SD, significant difference was tested by Student's *t* test. *P < .05, **P < .01, ***P < .001. ns = not significant. *P*‐value <.05 was considered as statistically significant

In order to investigate whether tigecycline‐inhibited cell proliferation was related to the cell cycle progression, cell cycle assay by virtue of flow cytometry was performed in cells treated with 5 and 10 μmol/L tigecycline, respectively, for 72 hours. The results revealed that the percentages of G0/G1 phase were dramatically increased in both AsPC‐1 and HPAC cells with tigecycline treatment, compared with that of the control groups (Figure 2A, B). It meant that tigecycline caused cell cycle arrest at G0/G1 phase in PDAC cells. To verify these results, we further examined cell cycle‐related proteins including CDK2, CDK4 and Cyclin E2 (CCNE2) in AsPC‐1 and HPAC cells, after treated with different concentrations of tigecycline for 72 hours. The results revealed that CDK2, CDK4 and CCNE2 were decreased after tigecycline treatment in a dose‐dependent manner (Figure 2C, D). Then, we also examined the G1 phase proteins in AsPC‐1 and HPAC cells after treated with 5 and 10 μmol/L tigecycline, respectively, for 0, 24, 48 and 72 hours. Interestingly, we found that CDK2, CDK4 and Cyclin E2 were also decreased in a time‐dependent manner (Figure 2E, F). These results showed that tigecycline could induce cell cycle arrest at G0/G1 phase in human PDAC cells in a dose‐ and time‐dependent manner.

### 3.3 Tigecycline inhibits cell migration and invasion in PDAC cells

**Figure 3:**
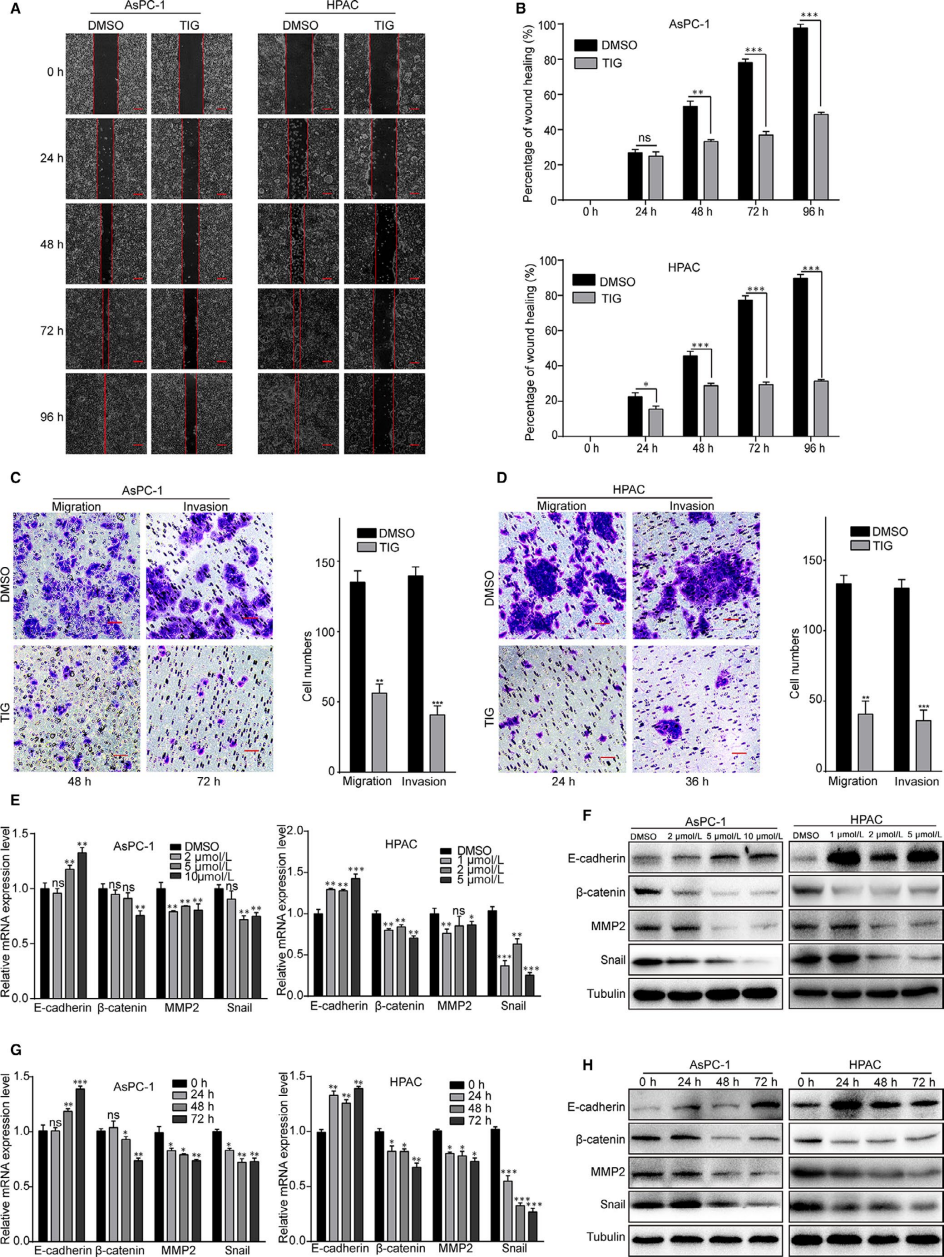
Tigecycline inhibits the cell migration and invasion in human PDAC cell lines. A, The migration by wound healing assay of AsPC‐1 and HPAC cells after treating with DMSO or IC_50_ of tigecycline for the indicated time. Scale bar, 100 μm. B, The effect of tigecycline on the wound closure in AsPC‐1 and HPAC cells. All data are shown as the mean ± SD. Student's *t* test was carried out. *P < .05; **P < .01; ***P < .001. *P*‐value <.05 was considered as statistically significant. C, D, Image and quantification of migration and invasion of AsPC‐1 and HPAC cells after treating with DMSO or 5 and 10 μmol/L tigecycline, respectively, for the indicated time. Scale bar = 100 μm. E, F, The expression of EMT‐related mRNA and proteins, E‐cadherin, β‐catenin, MMP2 and Snail in AsPC‐1 and HPAC cells treated tigecycline in different concentrations for 72 h. Tubulin was used as control. G, H, The expression of EMT‐related mRNA and proteins in AsPC‐1 and HPAC cells treated with DMSO or 5 and 10 μmol/L tigecycline, respectively, in different time for 0, 24, 48 and 72 h. Tubulin was used as control. All experiments were repeated at least three times. All data were shown as the mean ± SD. Student's *t* test was carried out. *P < .05; **P < .01; ***P < .001. ns, not significant. *P*‐value <.05 was considered as statistically significant

As one of the most malignant types of digestive tract cancer cells, PDAC cells showed powerful migration and invasion capabilities. In this study, the effect of tigecycline on cell migration and invasion abilities was investigated. Wound healing assay showed that cells treated with 5 and 10 μmol/L tigecycline, respectively, for 0, 24, 48, 72 and 96 hours displayed a significantly lower lateral migration rate into a wound introduced in a confluent monolayer of cells than that of the control groups (Figure 3A, B). In addition, transwell migration assay further revealed that PDAC cells after tigecycline treatment showed a seriously inhibition in cell migration ability, compared with that of the control groups (Figure 3C, D). Consistently, we further verified that PDAC cells treated with tigecycline prominently decreased invasive abilities to get through the Matrigel‐coated membrane, compared with controls by using transwell invasion assay (Figure 3E, F). Furthermore, consistent with above, Western blot indicated that cells treated in different concentrations tigecycline showed down‐regulated mesenchymal markers, such as β‐catenin, MMP2 and Snail, and an up‐regulated epithelial marker, E‐cadherin (Figure 3G, H). Then, we also examined the expression of epithelial‐mesenchymal transition (EMT) markers in cells treated with 5 and 10 μmol/L tigecycline, respectively, for 0, 24, 48 and 72 hours (Figure 3G, H). In summary, these results indicated that tigecycline inhibited cell migration and invasion in human PDAC cells.

### 3.4 Tigecycline enhances chemosensitivity to gemcitabine of PDAC cells in vitro

To investigate the role of tigecycline in apoptosis in PDAC cells, we performed flow cytometry in cells treated with 5 and 10 μmol/L tigecycline, respectively, and 1 μmol/L doxorubicin (DOX) as positive control for 72 hours. The results showed that cells treated with tigecycline had no significant or slight increase in apoptosis (Figure S2A, B). Consistently, Western blot was used to evaluate the cleavage of caspase‐3 (C‐caspase‐3) and PARP (C‐PARP), and the results displayed that C‐caspase‐3 and C‐PARP had no significant increase after tigecycline treatment, compared with DMSO groups (Figure S2C). These data suggested that tigecycline had little effect on apoptosis in PDAC cells.

**Figure 4:**
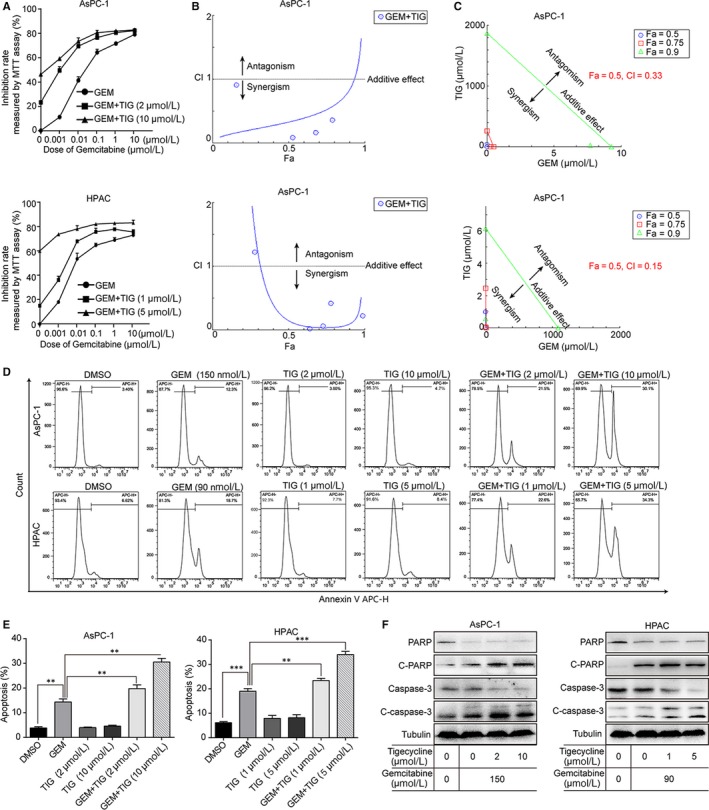
Tigecycline enhances chemosensitivity to gemcitabine in vitro. A, AsPC‐1 and HPAC cells were treated with increasing concentrations of gemcitabine and tigecycline for 72 h, and then, inhibition rates were measured by using MTT assay. B, C, The combination effect of tigecycline and gemcitabine in PDAC cells was measured by using MTT assay and calculated by using the CompuSyn software. D, E, Image and quantification of apoptosis rates of AsPC‐1 and HAPC cells after treating with IC_50_ of gemcitabine and increasing concentrations of tigecycline for 72 h. by flow cytometry. Statistical analysis of apoptosis intensity in treated cells was performed in vitro. F, AsPC‐1 and HPAC cells were treated with IC_50_ of gemcitabine, and increasing concentrations of tigecycline for 72 h, and then, the expression of cell apoptosis‐related proteins, cleaved caspase‐3, cleaved PARP and p53 were detected using Western blot assay. DMSO was used as control. Tubulin was used as control. All experiments were repeated at least three times. All data were shown as the mean ± SD. Student's *t* test was carried out. **P < .01; ***P < .001. *P*‐value <.05 was considered as statistically significant [Correction Statement: Correction added on xx March 2020 after first online publication: Figure 4 has been updated in this version.]

Furthermore, MTT assay was performed to determine the effect of tigecycline on chemosensitivity to gemcitabine (Figure S2D) in PDAC cells. The results displayed that combined treatment showed a more inhibitory effect on both AsPC‐1 and HPAC cells either at low concentrations or at high concentrations tigecycline (Figure 4A). Subsequently, we used CompuSyn to calculate the Combination Index (CI) at Fa = 0.5, and the results showed that tigecycline and gemcitabine synergically inhibits cell viability of PDAC cells (Figure 4B, C). Then, we also evaluated the role of cell apoptosis induced by combined treatment. The results revealed that apoptotic rates were significantly increased in PDAC cells treated with increasing concentrations of tigecycline combined with gemcitabine (Figure 4D, E). Consistently, IC_50_ of gemcitabine treatment alone up‐regulates the C‐caspase‐3 and C‐PARP levels in PDAC cells, which were also promoted by combining with tigecycline (Figure 4F). These data suggested that tigecycline could enhance chemosensitivity of PDAC cells to gemcitabine via inducing cell apoptosis.

### 3.5 The effect of tigecycline alone or combined with gemcitabine on the growth of PDAC xenografts

**Figure 5:**
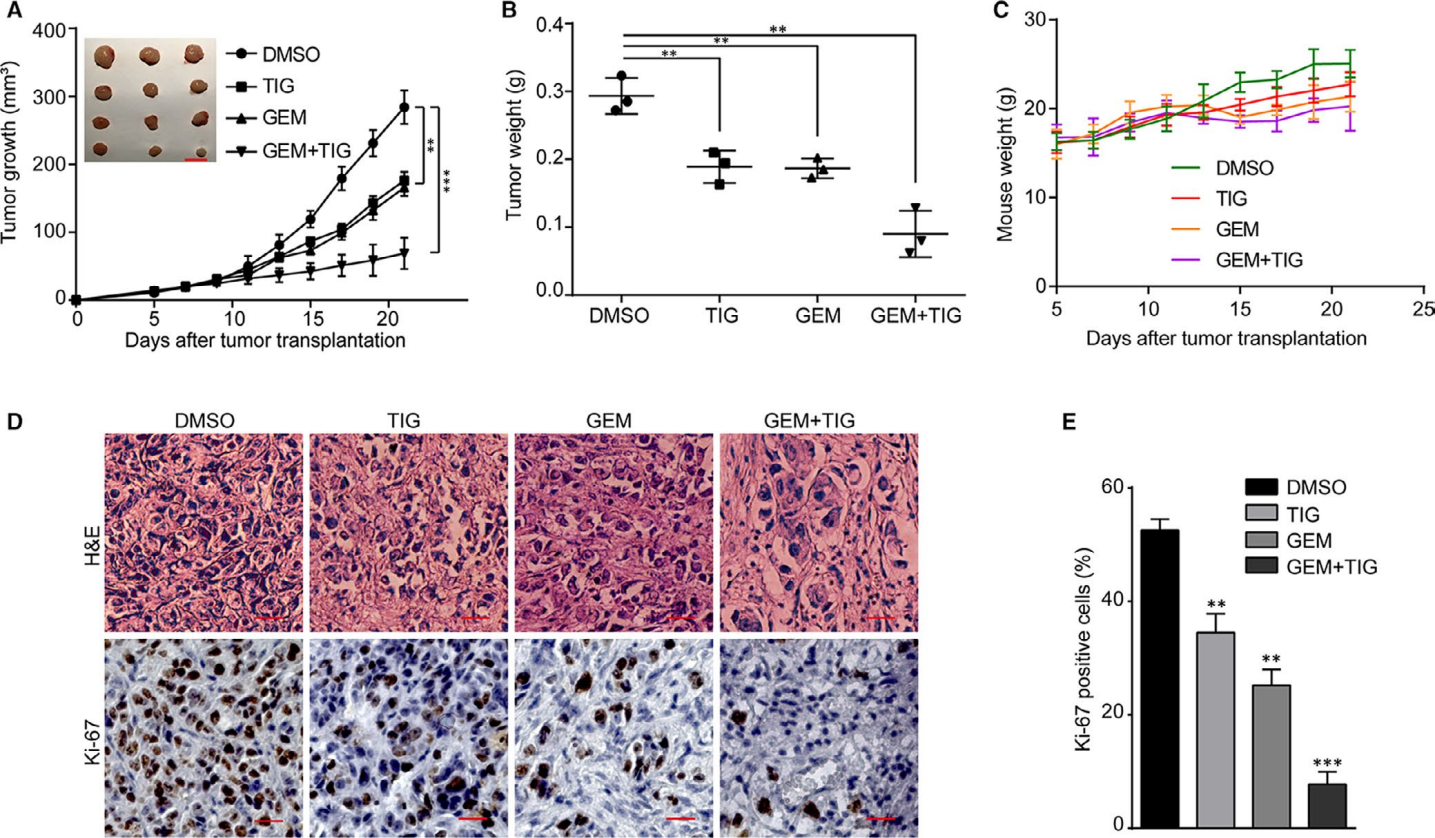
Tigecycline inhibits tumorigenicity and enhances chemosensitivity to gemcitabine in vivo. A, The tumour weights of the xenograft tumours in each group. B, The tumour volumes of the xenograft in each group, Scale bar: 1 cm, and the in vivo growth curves of xenograft tumours treated with tigecycline, gemcitabine alone and combined with each other. C, The weight of mice body was measured in each group. D, Haematoxylin and eosin (H&E) staining and immunohistochemical staining for Ki67 were performed. Scale bar = 100 μm. E, Effects of tumours on cell proliferation (Ki67) treated with tigecycline, gemcitabine alone and combined with each other in AsPC‐1 cells. All experiments were repeated at least three times. All data were shown as the mean ± SD. Student's *t* test was carried out. **P < .01; ***P < .001. *P*‐value <.05 was considered as statistically significant

To further assess the inhibition of tigecycline on human PDAC cells in vivo, AsPC‐1 cells were injected subcutaneously into nude mice to establish xenograft models for in vivo experiments. Then, the nude mice were randomly divided into four different groups: the control group, the tigecycline group (100 mg/kg), the gemcitabine group (50 mg/kg) and the combined treatment group. All the mice were killed and dissected on day 21 after different treatment, and the formed tumours were removed and weighed (Figure 5A). The results revealed that tigecycline or gemcitabine treatment significantly blocked tumour growth, especially, and the effect of combined treatment was the most significant (Figure 5B). However, there is no significant weight loss in tigecycline or gemcitabine or both treatment groups, compared with the control group (Figure 5C). Furthermore, IHC staining demonstrated that Ki‐67 expression was dramatically decreased in tigecycline‐ or gemcitabine‐treated cells, and combined treatment further decreased Ki‐67–positive percentages (Figure 5D, E). These data showed that tigecycline could effectively inhibit the development of tumours in vivo and the combination of gemcitabine and tigecycline exhibited a more significant inhibitory effect on tumorigenecity than that of the groups treated with gemcitabine alone.

### 3.6 CCNE2 is correlated with clinical outcome of patients with pancreatic adenocarcinoma

**Figure 6:**
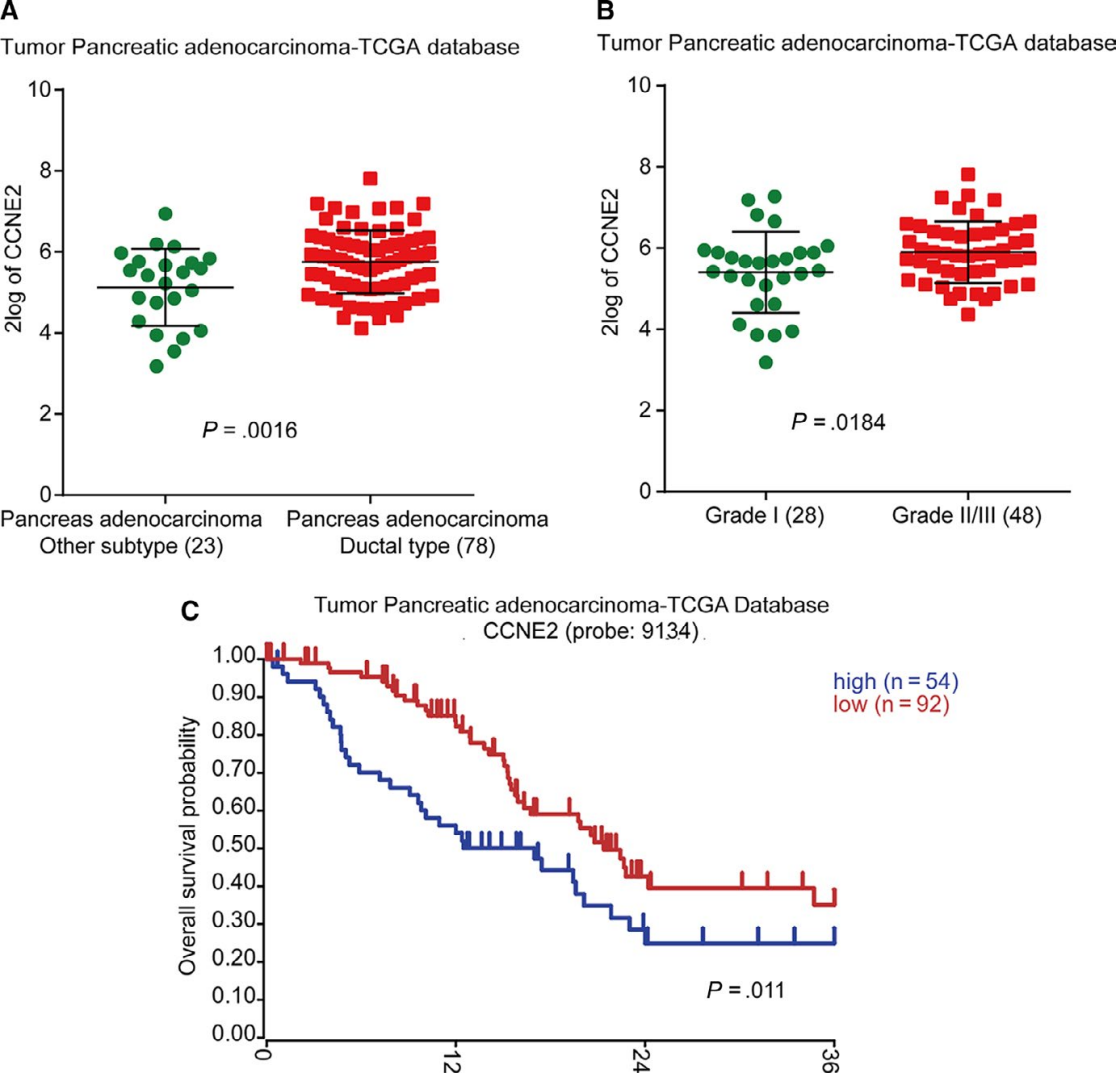
CCNE2 is correlated with clinical outcome of patients with pancreatic adenocarcinoma. A, The expression of CCNE2 in different subtypes of pancreatic adenocarcinoma in the TCGA database. B, The expression of CCNE2 in different grades of pancreatic adenocarcinoma in the TCGA database. C, Overall survival analysis for CCNE2 expression in pancreatic adenocarcinoma in the TCGA database

During the experiment, after treated with tigecycline in a dose‐ and time‐dependent manner, we found the expression of CCNE2, which was a G1 phase key regulatory factor, was significant decreased in both mRNA and protein levels (Figure 2). These data indicated that CCNE2 might play a potent role in tigecycline‐induced inhibition of cell proliferation. To confirm this hypothesis, we analysed the correlations of CCNE2 expression and clinical outcome of patients with pancreatic adenocarcinoma in the TCGA database. The result showed that CCNE2 was specifically up‐regulated in ductal type of pancreatic adenocarcinoma, compared with that of other types of pancreatic adenocarcinoma (*P* = .0016, Figure 6A). Besides, CCNE2 expression was also significantly higher in pancreatic adenocarcinoma of grade II/III than that of pancreatic adenocarcinoma of grade I (*P* = .0184, Figure 6B). Importantly, CCNE2 expression was negatively correlated with prognosis of overall survival probability of patients with pancreatic adenocarcinoma (*P* = .011, Figure 6C). All these results indicated that CCNE2 was correlated with clinical outcome of patients with pancreatic adenocarcinoma and might be a possible therapeutic target in the treatment of pancreatic adenocarcinoma.

### 3.7 Overexpression of CCNE2 rescues tigecycline‐induced cell proliferation inhibition and cell cycle arrest in PDAC cells

**Figure 7:**
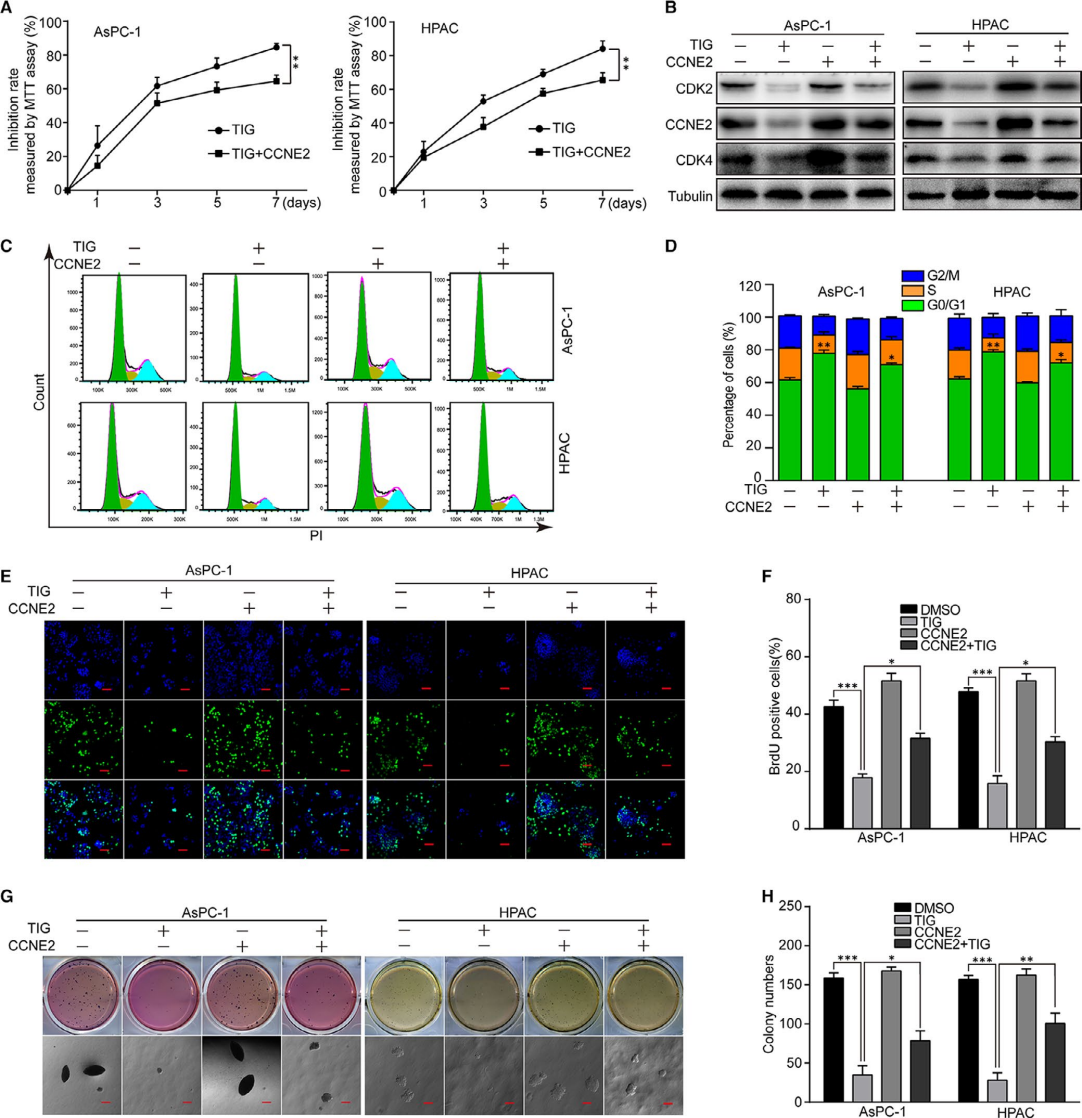
Overexpression of Cyclin E2 rescues tigecycline‐induced cell proliferation inhibition in human PDAC cell lines. A, Inhibition rates of CCNE2/vector‐overexpressed AsPC‐1 and HPAC cells after treating with 5 and 10 μmol/L tigecycline, respectively, for the indicated time using MTT assay. B, Western blot assay was used to show the expression of CCNE2, CDK2 and CDK4 after tigecycline treatment. Tubulin was used as control. C, D, The cell cycle was analysed by flow cytometry in CCNE2/vector‐overexpressed AsPC‐1 and HPAC cells after treated with 5 and 10 μmol/L tigecycline, respectively, for 72 h. E, F, Image and quantification of CCNE2/vector‐overexpressed AsPC‐1 and HPAC cells positive for BrdU staining after treating with 5 and 10 μmol/L tigecycline, respectively, for 72 h. Scale bar: 100 μm. G, H, Colony formation was examined by soft agar assay (1000 cells/well) in CCNE2/vector‐overexpressed AsPC‐1 and HPAC cells after treating with 5 and 10 μmol/L tigecycline, respectively, for 21‐28 d. Scale bar = 100 μm. Colony numbers were counted. All experiments were repeated at least three times. All data were shown as the mean ± SD. Student's *t* test was carried out. *P < .05; **P < .01; ***P < .001. *P*‐value <.05 was considered as statistically significant

AsPC‐1 and HPAC cells were stably infected with lenti‐virus encoding CCNE2, and Western blot revealed that CCNE2 expression was recovered after infection (Figure 7B). MTT assay was employed to investigate cell growth curve in CCNE2/vector‐overexpressed PDAC cells after treating with DMSO or 5 and 10 μmol/L tigecycline, respectively, for 7 days. The results showed that sensitivity to tigecycline in CCNE2‐overexpressed cells was lower than that in the vector‐overexpressed cells (Figure 7A). Moreover, Western blot also revealed that two other proteins CDK2 and CDK4 were also recovered by CCNE2 overexpression (Figure 7B). Therefore, we performed flow cytometry in CCNE2/vector‐overexpressed cells treated with 5 and 10 μmol/L tigecycline, respectively, for 3 days. The results showed that G1 arrest was also recovered by CCNE2 overexpression in tigecycline‐treated PDAC cells (Figure 7C, D). Next, BrdU and soft agar assay were also used to detect whether CCNE2‐overexpressed cells could restore cell proliferation and colony formative ability in PDAC cells. The results showed that cell proliferation inhibition and colony formative ability suppression were rescued after CCNE2 overexpression in tigecycline‐treated cells, compared with controls (Figure 7E‐H).

### 3.8 Overexpression of CCNE2 retrieves tigecycline‐induced cell migration and invasion suppression in human PDAC cells

**Figure 8:**
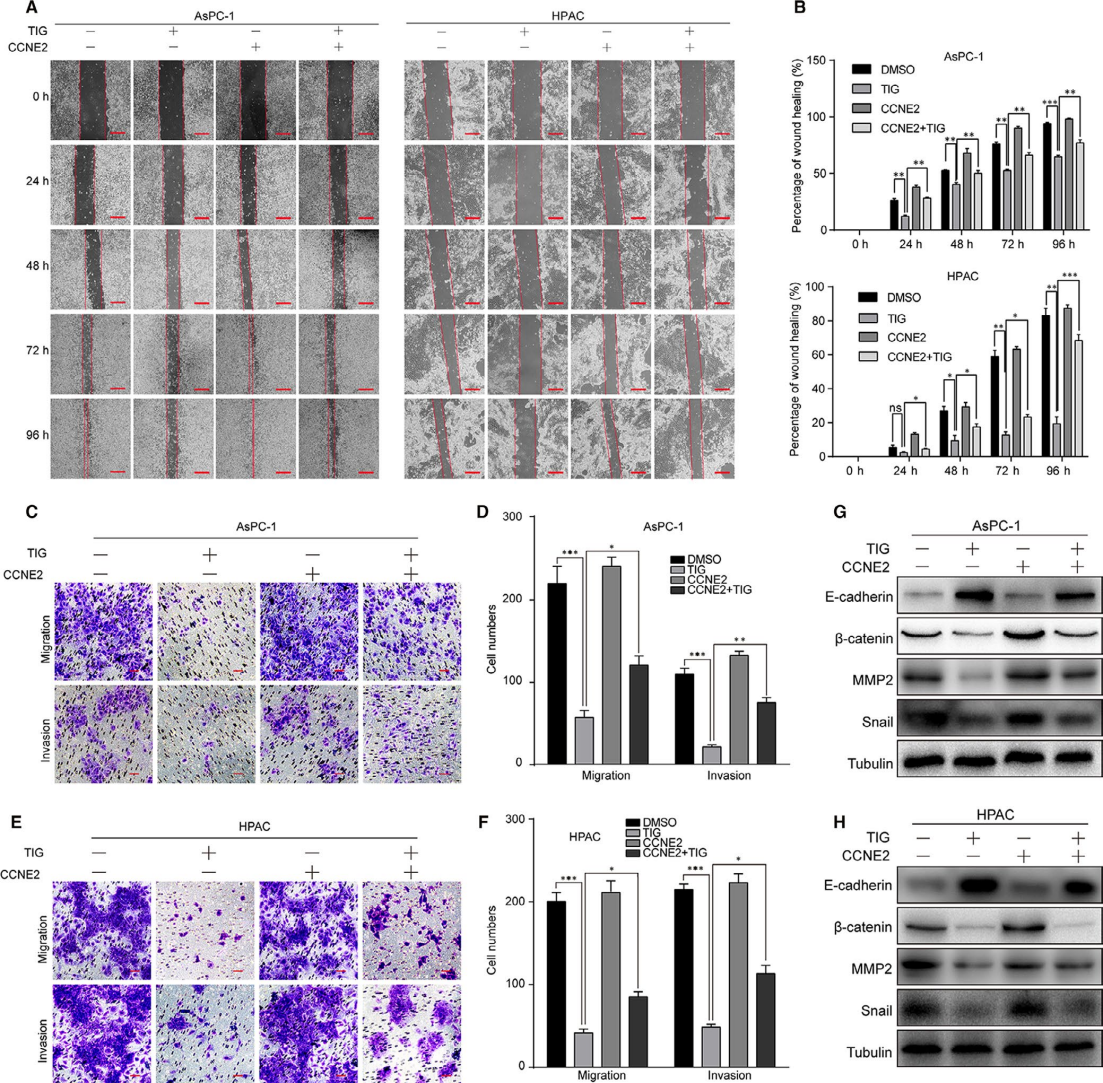
Overexpression of CCNE2 retrieves tigecycline‐induced cell migration and invasion suppression in human PDAC cell lines. A, B, The migration by wound healing assay in CCNE2/vector‐overexpressed AsPC‐1 and HPAC cells after treating with DMSO or IC_50_ of tigecycline for the indicated time. Scale bar, 100 μm. C, D, The effect of migration and invasion assays in CCNE2/vector‐overexpressed AsPC‐1 cells after treating with 10 μM tigecycline for 48 and 72 h, respectively. Scale bar: 100 μm. Migration and invasion cell numbers were counted. E, F, The effect of migration and invasion assays in CCNE2/vector‐overexpressed HPAC cells after treating with 5 μmol/L tigecycline for 36 and 48 h, respectively. Scale bar = 100 μm. Migration and invasion cell numbers were counted. (G, H) Western blot assay was used to show the expression of EMT‐related proteins in CCNE2/vector‐overexpressed AsPC‐1 and HPAC cells after treated with 5 and 10 μmol/L tigecycline for 72 h. Tubulin was used as control. All experiments were repeated at least three times. All data were shown as the mean ± SD. Student's *t* test was carried out. *P < .05; **P < .01; ***P < .001. *P*‐value <.05 was considered as statistically significant

As previously reported,^38^ CCNE2 was also reported to be tightly related to cell migration and invasion. Therefore, we further investigated the effect of cell migration in AsPC‐1 and HPAC cells by overexpressing CCNE2. Our data revealed the cell migration and invasion ability were rescued, at least partly, after CCNE2 overexpression in tigecycline‐treated cells, compared with that of the tigecycline‐treated vector groups (Figure 8A‐F). Simultaneously, we also measured the migration/invasion‐related proteins in CCNE2‐transfected cells after tigecycline treatment. Restoration of CCNE2 significantly rescued tigecycline‐induced increase in E‐cadherin expression and decrease in MMP2, β‐catenin and Snail expression (Figure 8G, H). Collectively, the results indicated that tigecycline‐induced migration/invasion inhibition was recovered by CCNE2 overexpression in PDAC cells.

## 4 DISCUSSION

As the first member of glycylcycline antibiotic drug, tigecycline was originally approved mainly used to treat bacterium infection by FDA.^14^ However, over the past few years, many researches demonstrated it effectively inhibited tumour development both in leukaemia and solid tumours.^16^ In the current study, we demonstrated that tigecycline inhibits cell proliferation, migration and invasion and leads to cell cycle arrest in PDAC cells. Besides, tigecycline suppresses tumorigenecity alone or combination with gemcitabine in vivo. All these results indicated that tigecycline shows a potential to treat patients with PDAC.

Our groups previously reported that tigecycline affected cell cycle progression in multiple malignant tumours, including melanoma,^20^ glioma,^22^ neuroblastoma 21 and oral squamous cell carcinoma.^19^ Consistently, tigecycline was also shown to be responsible for G0/G1 cell cycle arrest in PDAC cells by down‐regulating the expressions of Cyclin E2, CDK2 and CDK4. Besides, tigecycline inhibited cell migration and invasion in PDAC cells by down‐regulating mesenchymal markers, such as β‐catenin and Snail, and upregulating epithelial marker, E‐cadherin. Previously, we also found that tigecycline inhibited cell metastasis in melanoma via blocking the EMT.^20^ Tigecycline was shown to induce intrinsic caspase‐dependent apoptosis in ALL,^30^ retinoblastoma,^39^ cervical squamous cell carcinoma 28 and NSCLC.^24^ However, tigecycline did not cause significant apoptosis in PDAC cells when tigecycline was used alone. In consistence with our results, tigecycline did not cause apoptosis in both oral squamous cell carcinoma, glioma and gastric cancer either, as reported by our groups.^18^, ^19^, ^22^

Surprisingly, we found that tigecycline combined with gemcitabine enhances PDAC cellular chemosensitivity both in vitro and in vivo. When combined with tigecycline, the IC_50_ of gemcitabine in PDAC cell lines was remarkably declined. Besides, the combination treatment exerted a more effective inhibition in cancer cell growth both in vitro and in vivo, compared with gemcitabine or tigecycline treatment alone. Importantly, cleaved PARP, cleaved caspase‐3 and apoptotic rate were not up‐regulated when treating tigecycline alone in PDAC cells, but were up‐regulated significantly when treating tigecycline combined with gemcitabine in PDAC cells both in vitro and in vivo. These results implied that tigecycline augments the effect of chemotherapeutic treatment of PDAC. Consistently, previous reports also showed that tigecycline treatment could also promote chemosensitivity of many other tumours. For example, preclinical studies showed that tigecycline and venetoclax synergically inhibit MYC/BCL‐2 double‐hit B‐cell lymphoma.^40^ Besides, tigecycline dramatically improved the efficacy of doxorubicin and vincristine in ALL in another preclinical study.^30^ Combination treatment with imatinib and tigecycline selectively eradicated CML leukaemic stem cells both in vitro and in vivo.^31^ Tigecycline combined with paclitaxel significantly enhances therapeutic efficacy of renal cell carcinoma in vitro and in vivo.^41^ Tigecycline also significantly promoted the inhibitory effects of cisplatin in hepatocellular carcinoma in vitro and in vivo. All these results showed an excellent synergic role in augmenting of chemosensitivity in numerous tumours.

As tigecycline is an antibiotic drug, many groups showed that tigecycline played an important role in the function of mitochondria. Initially, tigecycline was shown to impair mitochondrial translation in AML,^17^, ^42^ renal cell carcinoma 41 and B‐cell lymphoma.^26^, ^43^ Besides, tigecycline inhibited mitochondrial oxidative phosphorylation to restrict energy and/or oxidative stress and damage in chronic myeloid leukaemia stem cells,^31^ acute lymphoblastic leukaemia (ALL),^30^ non–small‐cell lung cancer (NSCLC) 24 and hepatocellular carcinoma.^27^ However, tigecycline might have other targets in the cells except for proteins in mitochondria. For example, AKT signalling pathway (including its downstream factors p21 and mTOR) and Wnt/β‐catenin signalling are also potential targets of tigecycline.^16^

To explore the biological mechanism of tigecycline inhibiting PDAC cells’ growth and metastasis, we found that CCNE2, a target of p21, was significantly reduced in mRNA as the same to protein expression levels. As studies had pointed out that CCNE2 was not only related to cell cycle progression, but also related to cell metastasis,^38^, ^44^, ^45^, ^46^ we suggested that CCNE2 played a key role in PDAC cell treatment with tigecycline. Interestingly, CCNE2 expression was negatively correlated with clinical outcomes and grades of pancreatic adenocarcinoma. Therefore, we stably constructed CCNE2‐overexpressed PDAC cells. Then, we discovered that the cell cycle arrest at G0/G1 phase was dramatically weakened in CCNE2 overexpressed PDAC cell treatment with tigecycline, and the expression of cell cycle‐related proteins CCNE2, CDK2 and CDK4 was also recovered. Previous reports also showed that CCNE2 could drive tumour cell proliferation by activating its kinase partner CDK2,^47^, ^48^ but whether CCNE2 could directly regulate CDK4 was still unclear or CCNE2 affect the expression of CDK4 via an indirect manner. In addition, the ability to migration and invasion was also strengthened and expression of metastasis‐related proteins E‐cadherin, MMP2 was also recovered, but the expression levels of proteins β‐catenin and Snail were not significantly recovered. These results indicated that CCNE2 could also drive tumour cell migration and invasion by regulating metastasis‐related proteins as previously reported.^38^, ^49^

In summary, our data showed the function of tigecycline in human PDAC cells. We first demonstrated that tigecycline inhibited cell proliferation of PDAC cells, but interestingly, it had no effect on normal pancreatic duct glandular cells within a certain concentration range. Then, we selected two cell lines AsPC‐1 and HPAC cells to treat with tigecycline and found tigecycline‐induced cell cycle arrest and suppressed migration/invasion by down‐regulating CCNE2 expression, but could not induce apoptosis. In addition, tigecycline combined with gemcitabine increased apoptotic rates in PDAC cells, which meant that tigecycline enhanced the sensitivity of gemcitabine chemotherapy. Besides, CCNE2 overexpression could block the effects of cell cycle arrest and migration/invasion suppression induced by tigecycline. Furthermore, tigecycline effectively inhibited the proliferation and metastasis of PDAC cells. These findings suggested that tigecycline might be a promising drug candidate for the treatment of PDAC.

## CONFLICT OF INTEREST

The authors declare no conflict of interest.

## AUTHOR CONTRIBUTIONS

ZD and HC designed the experiments. JY, ZD, KZ and CL conducted the experiments, data acquisition and analysis. GF, AR, XW and KZ provided technical supports. ZD and J.Y wrote the manuscript. HC and XW revised the manuscript.

## Data Availability

All data included in this study are available upon reasonable request by contact with the corresponding author.
