# Supplementary material for: Antibiotic tigecycline inhibits cell proliferation, migration and invasion via down‐regulating CCNE2 in pancreatic ductal adenocarcinoma
Source: J Cell Mol Med. 2020 Mar 6;24(7):4245–60. doi: 10.1111/jcmm.15086 (PMC7171345; doi:10.1111/jcmm.15086)
Supplement: Supplementary file 1 [file JCMM-24-4245-s001.docx]

**SUPPLEMENTARY FIGURES**


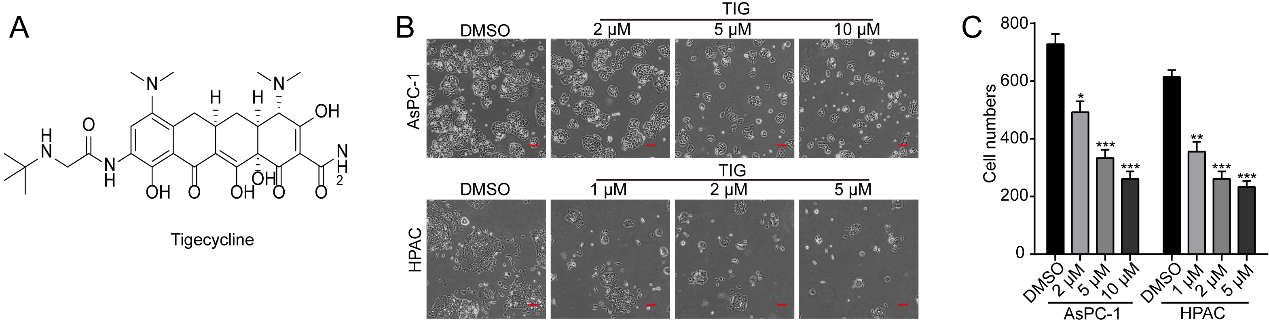


**Supplementary Figure S1. The chemical structure of tigecycline and its effect on proliferation in human PDAC cell lines.**

**(A)** The structure of Tigecycline. **(B)** The morphology of AsPC-1 and HPAC cells treating with different concentrations tigecycline for 72 h. Scale bar = 100 μm. **(C)** The quantification of AsPC-1 and HPAC cells treating with different concentrations tigecycline for 72 h. DMSO was used as control. All data are shown as the mean ± SD. Student’s t-test was carried out. *p < 0.05, **p < 0.01, ***p < 0.001. P-value <0.05 were considered as statistically significant.


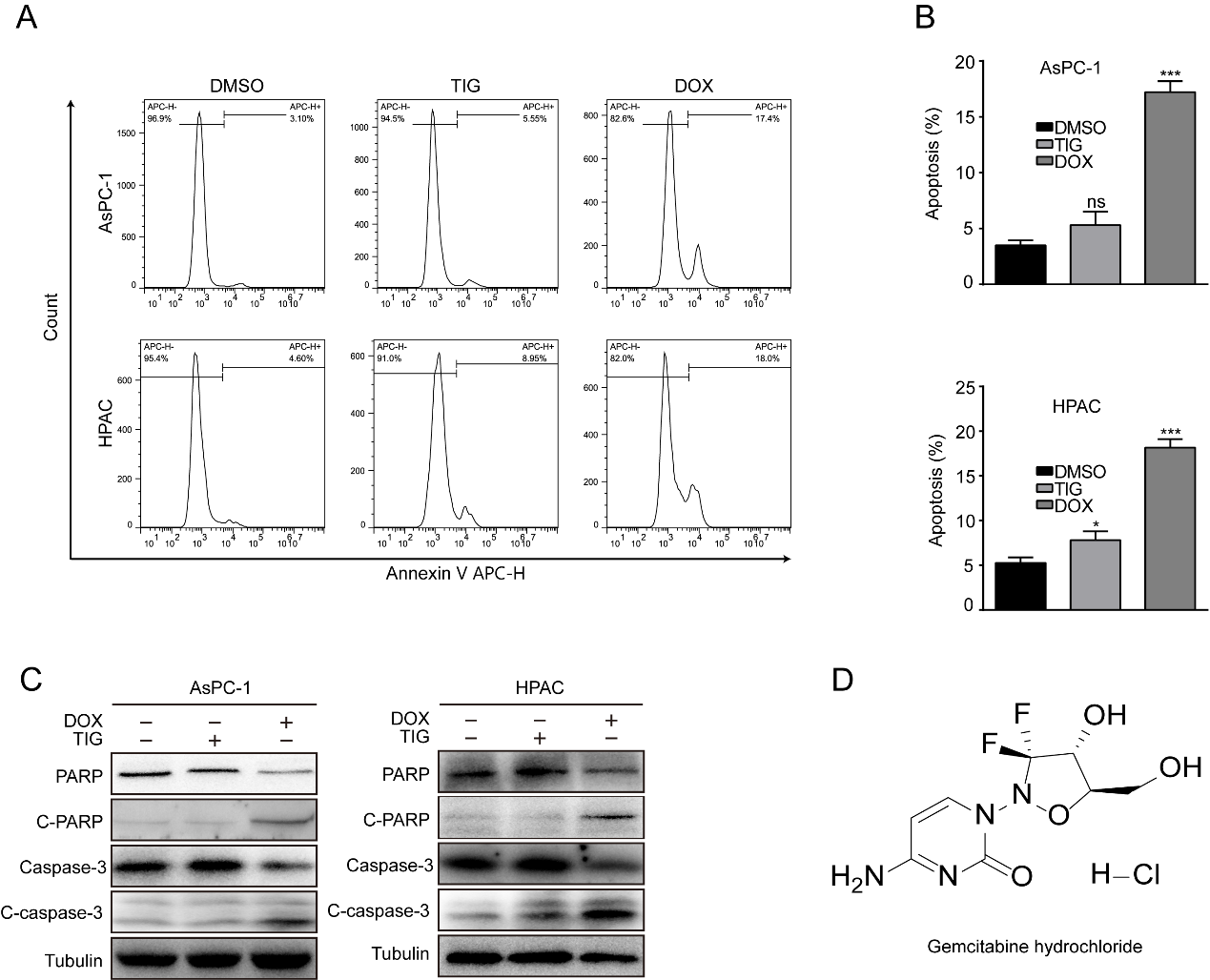


**Supplementary Figure S2. Tigecycline does not significantly induce apoptosis in human PDAC cell lines.**

**(A, B)** Image and quantification of apoptosis rates of AsPC-1 and HPAC cells after treating with DMSO or IC50 of tigecycline or 1μM doxycycline for 72 h by flow cytometry. Statistical analysis of apoptosis intensity in treated cells was not change in vitro. Doxycycline (DOX) was used as a positive control. **(C)** AsPC-1 and HPAC cells were treated with DMSO or IC50 of tigecycline or 1μM Doxycycline (DOX) for 72 h, and then the expression of cell apoptosis-related proteins, active Caspase-3, active PARP and P53 were detected using Western blot assay. Tubulin was used as control. **(D)** The structure of gemcitabine. All experiments were repeated at least three times. All data are shown as the mean ± SD. Student’s t-test was carried out. *p < 0.05, **p < 0.01, ***p < 0.001. P-value <0.05 were considered as statistically significant.
